# AMPK Modulates the Metabolic Adaptation of C6 Glioma Cells in Glucose-Deprived Conditions without Affecting Glutamate Transport

**DOI:** 10.3390/cells11111800

**Published:** 2022-05-31

**Authors:** Inês Belo do Nascimento, Marie Verfaillie, Gamze Ates, Pauline Beckers, Virginie Joris, Nathalie Desmet, Ann Massie, Emmanuel Hermans

**Affiliations:** 1 Institute of Neuroscience, Université Catholique de Louvain, 1200 Brussels, Belgium; ines.belo@uclouvain.be (I.B.d.N.); marie.verfaillie@uclouvain.be (M.V.); pauline.beckers@uclouvain.be (P.B.); nathalie.desmet@uclouvain.be (N.D.); 2 Center for Neurosciences, Neuro-Aging & Viro-Immunotherapy, Vrije Universiteit Brussel (VUB), 1090 Brussels, Belgium; gamze.ates@vub.be (G.A.); ann.massie@vub.be (A.M.); 3 Pole of Pharmacology and Therapeutics, Institute of Experimental and Clinical Research (IREC), Université Catholique de Louvain (UCLouvain), 1200 Brussels, Belgium; v.joris@maastrichtuniversity.nl

**Keywords:** ATP, astrocyte, metabolic stress, glucose deprivation, glutamate transporter

## Abstract

Energy homeostasis in the central nervous system largely depends on astrocytes, which provide metabolic support and protection to neurons. Astrocytes also ensure the clearance of extracellular glutamate through high-affinity transporters, which indirectly consume ATP. Considering the role of the AMP-activated protein kinase (AMPK) in the control of cell metabolism, we have examined its implication in the adaptation of astrocyte functions in response to a metabolic stress triggered by glucose deprivation. We genetically modified the astrocyte-like C6 cell line to silence AMPK activity by overexpressing a dominant negative mutant of its catalytic subunit. Upon glucose deprivation, we found that C6 cells maintain stable ATP levels and glutamate uptake capacity, highlighting their resilience during metabolic stress. In the same conditions, cells with silenced AMPK activity showed a reduction in motility, metabolic activity, and ATP levels, indicating that their adaptation to stress is compromised. The rate of ATP production remained, however, unchanged by AMPK silencing, suggesting that AMPK mostly influences energy consumption during stress conditions in these cells. Neither AMPK modulation nor prolonged glucose deprivation impaired glutamate uptake. Together, these results indicate that AMPK contributes to the adaptation of astrocyte metabolism triggered by metabolic stress, but not to the regulation of glutamate transport.

## 1. Introduction

Despite their high energy expenditure, neurons possess a limited reserve of energy substrates. They largely depend on the metabolic support provided by neighboring cells, in particular astrocytes, the adaptive properties of which appear to be critical to cope with energy demands during periods of intense synaptic activity [1,2]. Expressing a variety of receptors, transporters, and channels, astrocytes dynamically sense and respond to alterations in their microenvironment and the activity of nearby neurons. Ideally positioned at the blood–brain barrier and equipped with a robust enzymatic machinery for efficient glucose [3], glycogen [4], and lipid metabolism [5], astrocytes show the metabolic plasticity needed to support changes in nervous activity [1,6]. 

Astrocytes are also known as key supportive partners in the regulation of nervous transmission, as they buffer extracellular neurotransmitters, in particular glutamate. This is achieved through the activity of membrane high-affinity glutamate transporters that take up extracellular glutamate [7,8]. This efficient uptake is driven by the co-transport of Na^+^, which is pumped out of the cell by the Na^+^/K^+^-ATPase [9]. At low extracellular concentrations, glutamate is rapidly taken up, converted to glutamine, and transferred back to neurons to reconstitute presynaptic glutamate vesicles [10,11]. In addition, glutamate can also be used as an energetic fuel for both neurons and astrocytes [12]. In particular, at high extracellular concentrations, astrocytes efficiently use glutamate as an energetic substrate to produce ATP and support its uptake [13]. In contexts of intense excitatory activity, the astrocytic consumption of ATP is also compensated by a concomitant metabolic adaptation of astrocytes that promotes glucose uptake and glycolysis [14,15,16]. Thereby, besides their role in the clearance of glutamate, astrocytic glutamate transporters also act as sensors of neuronal activity, supporting the tight coupling between glutamate transmission and metabolism [1]. 

Recognized as the fuel gauge of the cell, adenosine monophosphate-activated protein kinase (AMPK) is the best documented cellular energy sensor [17,18]. Widely conserved among eukaryotes, AMPK is a heterotrimeric complex containing a catalytic alpha subunit (α) and two regulatory subunits, beta (β) and gamma (γ) [19,20,21,22]. This enzyme is mostly known for its ability to sense variations in the adenine nucleotide ratio (AMP/ATP) that reflects the energy status of cells. AMPK is activated by an imbalance in energy levels, which can be the consequence of various cellular insults, such as energetic substrate deprivation or hypoxia. In response to a stimulus, AMPK then regulates a wide array of cellular pathways, activating ATP-producing mechanisms while inhibiting ATP-consuming processes, and hence reestablishing energy homeostasis [23,24]. As a central actor in the control of cell metabolism, AMPK appears to be a key enzyme in tissues with high metabolic demands, such as the liver, heart, and muscle, but also in tumor cells, known for their altered metabolism [25]. Compared to these tissues, the expression and role of AMPK in astrocytes has so far received little attention. Early studies on cultured glial cells and brain samples suggested that the predominant AMPK activity in the brain parenchyma resides in astrocytes [26]. Later, Turnley and colleagues reported on the predominant expression of the AMPKα_2_ subunit in activated astrocytes within the cortex of dysmyelinated transgenic mice [27]. Further in vitro studies have identified the expression of both AMPKα isoforms in cultured astrocytes [28], where they contribute to the regulation of several aspects of astrocyte metabolism. Notably, AMPK activation in astrocytes increases glucose uptake and glycolysis, and has been suggested to increase its oxidation as well [29]. Recently, AMPK has also been shown to contribute to the regulation of astrocytic glycogen stores [30]. 

While a role for AMPK has been proposed in neurodegenerative disorders [31,32], the pivotal role of astrocytic AMPK for neuronal transmission and overall brain function has only recently been addressed. Indeed, Muraleedharan et al. showed that selective deletion of AMPK in astrocytes—but not in neurons—results in important neuronal loss and reduction of cortical thickness. In their study, AMPK-null astrocytes showed compromised glucose uptake, glycolysis, and lactate production, indicating that AMPK regulates the astrocyte–neuron lactate shuttle [33,34]. 

We hypothesized that AMPK is crucial for the adaptive properties of astrocytes upon cellular insult; hence, we sought to further characterize the involvement of AMPK in the control of astrocytic functions, and in particular, the regulation of glutamate uptake. We therefore used the C6 astrocytoma cell line [35], as a reliable astrocyte-like cell model that has been used for decades to study astrocyte biology [36,37,38]. We genetically modified this cell line to allow manipulation of AMPK activity through the overexpression of a dominant negative AMPKα_1_ isoform. The consequence of silencing AMPK activity on the metabolic phenotype of C6 cells, as well as their capacity to take up glutamate, was investigated in standard culture conditions, but also in response to a metabolic stress triggered by glucose deprivation. 

## 2. Materials and Methods

### 2.1. Generation of the Inducible AMPK-DN System

Dr. Rider (UCLouvain, Belgium) kindly provided the pcDNA3 vector containing a myc-tagged sequence encoding the dominant negative mutant (D157A) of the rat AMPKα_1_ subunit [39]. This sequence was subcloned in the pTRE2hyg vector (TakaraBio, Kusatsu, Japan), under the control of a tetracycline-inducible promoter.

### 2.2. Cell Transfection and Selection

C6 cells expressing the reverse Tet-responsive transcriptional activator (rtTA) were obtained as previously described [40] and were cultured in DMEM (Dulbecco’s Modified Eagle’s Medium 41965, ThermoFisher Scientific, Waltham, MA, USA) supplemented with 10% foetal bovine serum, 50 µg/mL penicillin-streptomycin, and 2.5 µg/mL amphotericin B (ThermoFisher Scientific), in a humidified atmosphere (5% CO_2_, 37 °C). C6-rtTA cells were transfected with the pTRE2hyg-AMPK-DN construct through the method of calcium phosphate precipitation [41]. After selection with hygromycine (300 µg/mL; InvivoGen, San Diego, CA, USA), clones were isolated and tested for AMPK-DN expression by myc detection after supplementing the culture medium with doxycycline (DOX; Takara Bio). The DOX induction protocol was then set at 2 µg/mL for 24 h.

### 2.3. Glucose Deprivation Protocol

The high-glucose (25 mM) culture medium (DMEM 41965, ThermoFisher Scientific) was replaced with low-glucose (0.1 mM) or glucose-free medium (DMEM 11966, ThermoFisher Scientific) for 3 and 6 h. Low-glucose medium was obtained by mixing high-glucose and glucose-free media. 

### 2.4. Total RNA Extraction and Real-Time Quantitative PCR (RT-qPCR)

Total RNA was extracted from cells using the TriPure isolation reagent (Sigma-Aldrich, Saint Louis, MO, USA) and reverse-transcribed using the iScript cDNA synthesis kit (Bio-Rad Laboratories, Hercules, CA, USA). Real-time PCR amplifications were performed on the Bio-Rad CFX Connect™ real-time PCR detection system (Bio-Rad Laboratories). Thirty-five cycles of amplification were carried out in a total volume of 20 μL, containing 27 ng of cDNA template, 0.5 μM of the appropriate primers, and the iTaq Universal SYBR Green Supermix, following the manufacturer’s instructions (Bio-Rad Laboratories). The absolute quantification of *Prkaa1* and *Prkaa2* sequences (respectively encoding for AMPKα_1_ and AMPKα_2_) was carried out using plasmid constructs containing the corresponding rat sequences as reference, and results were expressed as copy number per 10 ng of cDNA. For relative quantification, the analysis was performed using the delta-delta Ct method, where the amplified level of the targeted genes was normalized to that of the housekeeping gene glyceraldehyde 3-phosphate dehydrogenase (*Gapdh*). The sequences of the primers used in the RT-qPCR reactions are indicated in Table 1.

### 2.5. Western Blot

C6-rtTA-AMPK-DN cells were seeded into six-well plates at a density of 200,000 cells/well. Cells were scraped in ice-cold lysis buffer (50 mM Tris-HCl, 150 mM NaCl, 1 mM EGTA, 5mM EDTA, 0.1% sodium dodecyl sulfate (SDS), 0.5% sodium deoxycholate, 1% Igepal NP40) containing protease inhibitor cocktail (Halt™ Protease and Phosphatase Inhibitor, ThermoFisher Scientific) and a phosphatase inhibitor (PhoSTOP^TM^, Sigma-Aldrich). The protein concentration was determined using the Pierce™ BCA Protein Assay Kit (ThermoFisher Scientific). Equal amounts of protein (12 µg) diluted in a loading buffer (60 mM Tris-HCl pH 6.8, 10% glycerol, 5% β-mercaptoethanol, 2% SDS, and 0.01% bromophenol blue) were separated through SDS-polyacrylamide gel electrophoresis and transferred onto a nitrocellulose membrane. To avoid unspecific immunoprobing, membranes were blocked for 1 h in a Tris-buffered saline solution containing 0.05% Tween-20 (TBS-T) and 5% bovine serum albumin (Carl Roth). Immunoprobing was carried out overnight at 4 °C with primary antibodies recognizing the following proteins: phospho-ACC (1:2000; #3661S, Cell Signaling), ACC (1:1000; #3662S, Cell Signaling), c-Myc (1:1000; sc-789, Santa Cruz), AMPKα (1:5000; #2532S, Cell Signaling), and GAPDH (1:1,000,000; G9545, Sigma). After washing with TBS-T, membranes were incubated for 1 h at room temperature with the respective peroxidase-conjugated secondary antibodies (Jackson ImmunoResearch, Cambridgeshire, UK). Immunoreactivity was detected using Clarity enhanced chemiluminescence reagent (Bio-Rad Laboratories). Quantification of the signals was performed using ImageJ software (version 1.46r, Wayen Rasband, US National Institutes of Health, Bethesda, MD, USA).

### 2.6. d-[^3^H]-Aspartate Uptake

The activity of glutamate transporters was evaluated by uptake assays using radiolabeled d-aspartate as substrate (d-[^3^H]-aspartate), a transportable non-metabolized analogue of l-glutamate that does not interact with glutamate receptors. Cells were seeded into 24-well plates at a density of 30,000 cells/well. Multi-well plates were placed at the surface of a 37 °C water bath. The culture medium was removed, and the cells were rinsed three times with preheated Na^+^-containing Krebs buffer (25 mM HEPES pH 7.4, 4.8 mM KCl, 1.2 mM KH_2_PO_4_, 1.3 mM CaCl_2_, 1.2 mM MgSO_4_, and 120 mM NaCl). d-[^3^H]-aspartate (specific activity of 12.2 Ci/mmol, Perkin Elmer) was added to the cells at a final concentration of 50 nM. After 20 min, the uptake was stopped by three rinses with ice-cold Na^+^-free Krebs buffer (25 mM HEPES pH 7.4, 4.8 mM KCl, 1.2 mM KH_2_PO_4_, 1.3 mM CaCl_2_, 1.2 mM MgSO_4_, and 120 mM choline chloride), and cells were lysed with ice-cold 0.1 N NaOH. Radioactivity measurements were performed by mixing aliquots of the lysates with the liquid scintillation solution Microscint 40 and using the TopCount NXT Microplate Scintillation and Luminescence Counter (Perkin Elmer). Counts per minute were converted to pmol of d-[^3^H]-aspartate transported per min based on the specific activity of the radiolabeled substrate and the duration of the assay. In addition, aliquots of the lysates were used for protein quantification by the Bradford method using the BioRad Protein Assay Dye Reagent (BioRad Laboratories). The specific (glutamate-transporters-dependent) activity was calculated by subtracting the d-[^3^H]-aspartate uptake measured in the presence of the non-selective glutamate transporter inhibitor l-threo-3-hydroxyaspartic acid (100 μM LTHA, Tocris). Results were expressed as pmol of d-[^3^H]-aspartate transported per min per mg of protein.

### 2.7. MTT Assay

C6-rtTA-AMPK-DN cells were seeded into 96-well plates at a density of 10,000 cells/well. Following the appropriate treatments, to assess the cell metabolic activity, medium was replaced by 100 µL of 0.5 mg/mL 3-(4,5-dimethyl-2-thiazolyl)-2,5-diphenyl-2H-tetrazolium bromide (MTT; Sigma Aldrich) diluted in the culture medium with the same glucose concentrations. After a 2 h incubation period at 37 °C, the supernatant was discarded, and the reaction was stopped by adding a mixture of isopropanol/HCl 0.04 N. Absorbance was measured with a microplate reader (Victor X-3 Multilabel Plate Reader, Perkin Elmer, Waltham, MA, USA). For DOX-treated and non-treated cells, absorbance values in glucose-deprived conditions were converted as the percentage of the respective control condition (25 mM of glucose) and expressed as the difference with the latter.

### 2.8. ATP Assay

To measure the total ATP content of C6-rtTA-AMPK-DN cells, a luciferase-based method was used. Briefly, cells were seeded into opaque-walled 96-well plates at a density of 10,000 cells/well. Following the appropriate treatments, 100 µL of the CellTiter-Glo^®^ Reagent (Promega, Madison, WI, USA) was added to the wells. After gentle mixture and an incubation period of 10 min, relative luminescence units (RLU) were measured with the Victor X-3 Multilabel Plate Reader. For DOX-treated and non-treated cells, absorbance values in glucose-deprived conditions were converted as the percentage of the respective control condition (25 mM of glucose) and expressed as the difference with the latter.

### 2.9. XF Real-Time ATP Rate Assay

Cells were seeded at a density of 5000 cells/well in Seahorse-dedicated XF 96 Cell Culture Microplates (Agilent Technologies, Santa Clara, CA, USA). DOX treatment was initiated after overnight rest for 24 h, followed by exposure to different concentrations of glucose for 5 h. The XF Real-Time ATP Rate Assay (Agilent Technologies) was performed according to the manufacturer’s instructions. Briefly, cells were washed twice and incubated for 1 h with a complete DMEM-based XF medium (Agilent Technologies) containing 1 mM sodium pyruvate, 2 mM glutamine, and 0, 0.1, or 10 mM glucose (with and without DOX), in a non-CO_2_ incubator at 37 °C. After the overnight two-step hydration protocol of the sensor cartridge, ports A and B were loaded with the metabolic modulators oligomycin (15 μM) and rotenone/antimycin A (5 μM), to assure that the cells were exposed to a final concentration of 1.5 and 0.5 μM of the respective compounds. The Seahorse XFe96 flux analyzer (Agilent Technologies) was used to measure the oxygen consumption rate and the extracellular acidification rate, using the default protocol of the XF Real-Time ATP Rate Assay (three measurements in cycles of 5 min and automatic injection of oligomycin and rotenone/antimycin A after 18 min and 36 min, respectively). Data were normalized by automatic cell counting after staining with Hoechst 33342 (8 μM, 30 min incubation in the dark, Sigma-Aldrich), using the Cytation 1 (BioTek). Data quality control and initial analyses were performed using the Seahorse Analytics software (Agilent Technologies).

### 2.10. Migration Assay

C6-rtTA-AMPK-DN cells were seeded into 12-well plates at a density of 100,000 cells/well. After reaching confluence, cell monolayers were wounded using a 20 µL micropipette tip, washed with phosphate-buffered saline, and incubated with the appropriate fresh culture medium containing either 25 or 0 mM glucose. Pictures were first taken 8 h after wounding and then every 2 h over a period of 12 h, using the EVOS FL Auto 2 microscope (ThermoFisher Scientific). The area of the wound was quantified using ImageJ software, and the migration rate (µm/min) was calculated as described previously [42].

### 2.11. Statistics

Data were obtained from at least three biological replicates (independent experiments conducted on cells from different passages) and were expressed as means with the standard error of the mean (SEM). When mentioned in the figure caption, technical replicates within each experiment were also performed. Statistical analyses were conducted using GraphPad Prism (GraphPad Software, version 5.03, San Diego, CA, USA). When comparing two data sets, differences between groups were evaluated using paired Student’s *t*-tests. When comparing more than two groups, one-way ANOVA followed by a Dunnett’s post-hoc test was used. Two-way ANOVA followed by a Bonferroni’s multiple comparisons test were used in two-factor analyses. In all statistical analyses, a value of *p* < 0.05 was considered as significant.

## 3. Results

### 3.1. Expression of an AMPK Dominant Negative Mutant in C6 Cells

The influence of manipulating AMPK activity on the metabolic adaptation of C6 cells was studied in a cellular model that allows tight control of the expression level of a dominant negative mutant of the AMPKα_1_ subunit (α_1_DN). In detail, the cDNA sequence encoding the myc-tagged α_1_DN was introduced in C6 cells engineered to carry the Tet-On expression system (C6-rtTA cells). A stable cell clone, referred to as C6-rtTA-AMPK-DN, was selected, and the efficacy of the inducible system was examined by RT-qPCR.

**Figure 1:**
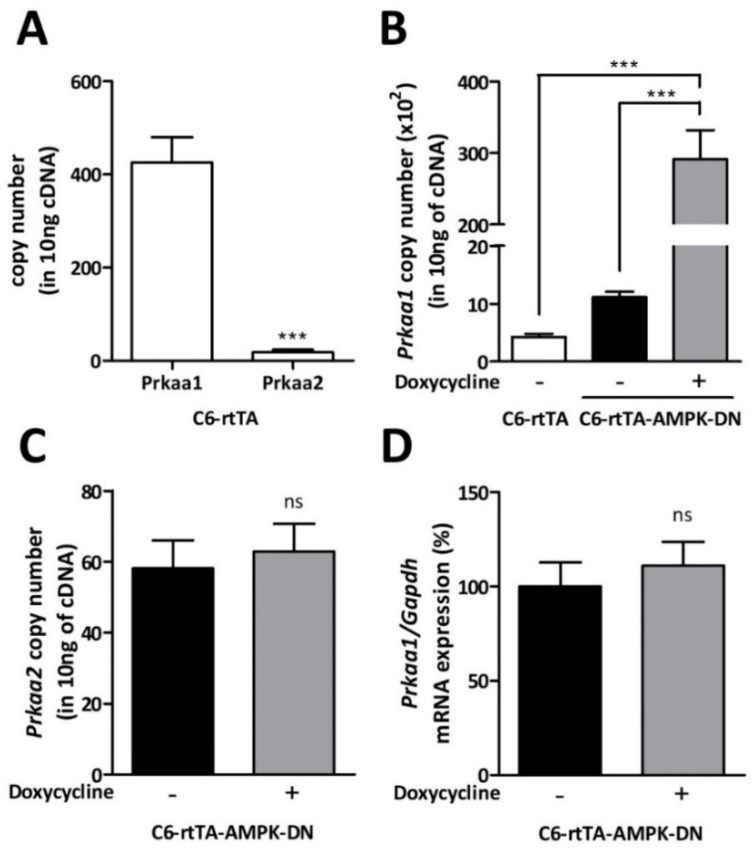
AMPKα mRNA expression in C6-rtTA and C6-rtTA-AMPK-DN cells. (**A**) The expression profile of AMPKα isoforms in C6-rtTA cells was quantified by RT-qPCR. Plasmid constructions carrying either the *Prkaa1* or *Prkaa2* coding sequences were used as standards for absolute quantification. (**B**,**C**) The mRNA expression of *Prkaa1* (endogenous and mutated combined) (**B**) and *Prkaa2* (**C**) was evaluated in C6-rtTA-AMPK-DN cells with or without DOX-induction. (**D**) The relative expression of the endogenous AMPKα_1_ isoform was evaluated in C6-rtTA-AMPK-DN cells induced or not with DOX, by using PCR primers targeting a non-coding sequence within the α_1_ sequence (cf. Table 1 for primer sequences). Data shown represent the mean ± SEM from four biological replicates. Statistical analyses were performed by paired Student’s *t*-tests (**A**,**C**,**D**) or one-way ANOVA followed by a Bonferroni’s multiple comparison test (**B**) (ns: non-significant; *** *p* < 0.001).

The mRNAs encoding for AMPKα_1_ and α_2_ were first quantified in C6-rtTA cells to characterize the endogenous expression of these isoforms. Plasmid constructs carrying either the *Prkaa1* or the *Prkaa2* coding sequences were used as standard for absolute quantification, and PCR primers targeting these coding sequences were designed. As shown in Figure 1A, C6-rtTA cells are characterized by a predominant expression of the α_1_ isoform compared to the α_2_ isoform (>20-fold difference). In C6-rtTA-AMPK-DN cells, the expression of the α_1_ isoform was considerably increased when exposed to doxycycline (DOX) for 24 h (30-fold increase) (Figure 1B), confirming the efficacy of the inducible system. In the same condition, the expression of the α_2_ isoform was not changed (Figure 1C; 58 and 63 copies/ng cDNA for control and DOX-induced cells, respectively). In order to demonstrate that the induction protocol was without influence on the endogenous expression of the α_1_ isoform (Figure 1D), PCR primers targeting the *Prkaa1* cDNA within the non-coding sequence were used (Table 1 for primer sequence). Indeed, this sequence is absent from the α_1_DN construct used for cell transfection.

**Figure 2:**
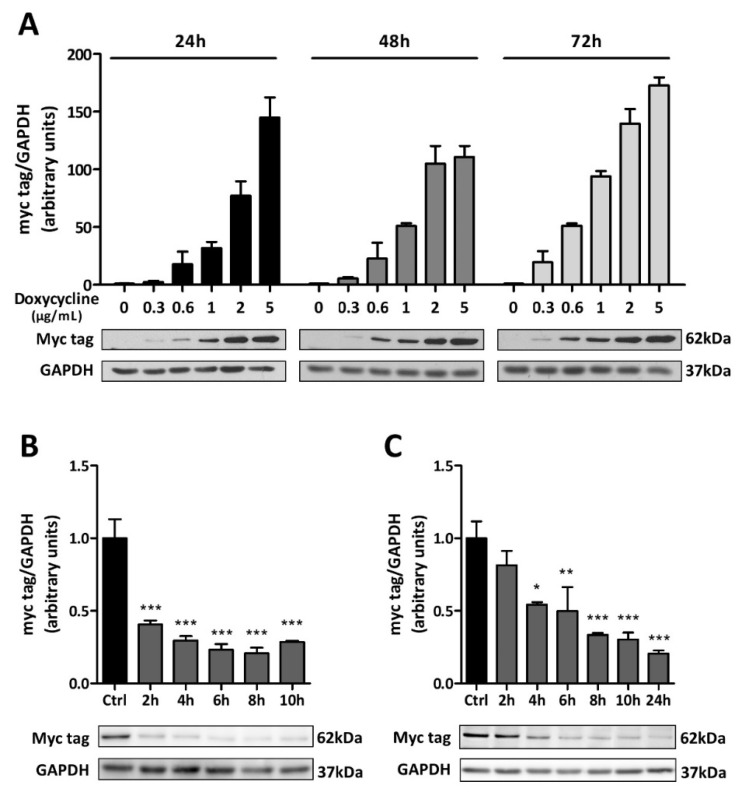
Validation of the inducible system. (**A**) C6-rtTA-AMPK-DN cells were exposed to increasing concentrations of DOX for 24, 48, and 72 h and the expression of the myc-tagged α_1_DN was evaluated by immunoblotting. (**B**,**C**) To assess for the stability of the α_1_DN, cells were either exposed to 10 µg/mL of cycloheximide (**B**), or the culture medium was renewed to remove DOX after 24 h (**C**). Cells were collected every 2 h, and the expression of the myc-tagged α_1_DN was evaluated by immunoblotting. Histograms show means ± SEM normalized to GAPDH expression. Blots shown are representative of three independent experiments. Blots in panel **C** were cropped to remove nonessential experimental conditions. Statistical analyses were performed by one-way ANOVA followed by Dunnett’s multiple comparison test (* *p* < 0.05, ** *p* < 0.01, *** *p* < 0.001).

The expression of the α_1_DN was further validated by immunoblotting after exposing C6-rtTA-AMPK-DN cells to DOX for 24, 48, or 72 h. As shown in Figure 2A, in the absence of DOX, no myc-tag immunoreactive signal could be detected at the expected molecular weight of AMPKα (62kDa). Exposure of the cells to DOX caused a concentration-dependent increase in the expression of the myc-tagged AMPK-DN, at all time-points tested. For the following experiments, the DOX-induction protocol was set at 2 µg/mL for 24 h.

To evaluate the stability of the α_1_DN upon induction with DOX, C6-rtTA-AMPK-DN cells were treated with cycloheximide (10 µg/mL), an inhibitor of protein synthesis. Considering the potential toxicity of this compound, the incubation period was limited to a maximum of 10 h. As shown in Figure 2B, after only 2 h of incubation with cycloheximide, the expression of the myc-tagged AMPK-DN was decreased by almost 60%, reaching its lowest level after 8 h. In line with these observations, removing DOX from the culture medium after the 24 h induction protocol also elicited a rapid reduction of the myc-immunoreactivity (Figure 2C). Taken together, these results indicate the rather short half-life of the α_1_DN, which was taken into consideration when designing the following experiments. In particular, it was ensured that DOX was present in the culture medium at all times, even throughout the glucose deprivation protocol.

### 3.2. Validation of the Doxycycline-Inducible AMPK-DN System

**Figure 3:**
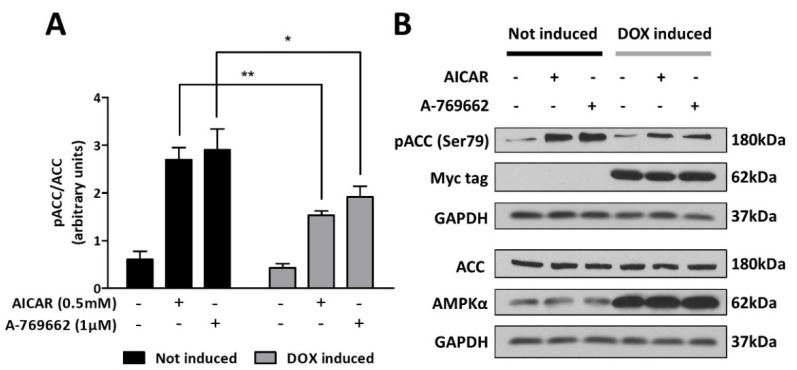
Silencing efficacy of the dominant negative mutant of AMPKα_1_. C6-rtTA-AMPK-DN cells, induced or not with DOX (2 µg/mL; 24 h), were exposed to the pharmacological AMPK activators AICAR (0.5 mM) and A-769662 (1 µM) for 3 h. AMPK activity was assessed by immunoblot analysis of the phosphorylation of its downstream target ACC. Phosphorylated ACC and total ACC were immunoblotted on separate gels, and their expression level was normalized to GAPDH. (**A**) Histograms show means ± SEM. (**B**) Blots shown are representative of six independent experiments. Statistical analyses were performed by two-way ANOVA followed by Bonferroni’s multiple comparison test (* *p* < 0.05, ** *p* < 0.01).

To determine the efficacy of the DN approach to silence AMPK activity, C6-rtTA-AMPK-DN cells cultured in the presence or absence of DOX were treated for 3 h with two distinct pharmacological AMPK activators: 5-aminoimidazole-4-carboxamide-1-β-D-ribofuranoside (AICAR; Toronto Research Chemicals; 0.5 mM dissolved in the culture medium) and compound A-769662 (1 µM; TOCRIS; pre-dissolved in DMSO—final concentration of DMSO in the culture medium of 0.001%). The expression of the α_1_DN upon induction was systematically verified by immunoblot and detection of the myc-tag immunoreactivity at the expected molecular weight of AMPKα, and by evaluating total AMPKα expression using an antibody that recognizes both the endogenous and mutated proteins. The AMPK activity was then assessed by immunoblot analysis of the phosphorylation of acetyl-CoA carboxylase (ACC), a well-known downstream target of AMPK, at serine 79 residue (Figure 3). In cells cultured in the absence of DOX, both AICAR and A-769662 elicited an increase in the pACC/ACC ratio (4.5-fold and 4.8-fold, respectively), thus reflecting a robust increase in AMPK activity.

Following DOX treatment, the basal levels of ACC phosphorylation remained unchanged, indicating that the overexpression of the mutated AMPK had no effect on the constitutive activity of AMPK. In contrast, in response to AICAR and A-769662, even though an increase in the pACC/ACC ratio was observed, it was shown to be 48 and 36% lower (respectively) compared to DOX-free conditions. Taken together, these results indicate that the overexpression of the α_1_DN ensures a partial but significant silencing of the activity of AMPK, when challenged with pharmacological activators. 

### 3.3. AMPK Silencing Limits the Metabolic Adaptation to Glucose Deprivation in C6 Cells

**Figure 4:**
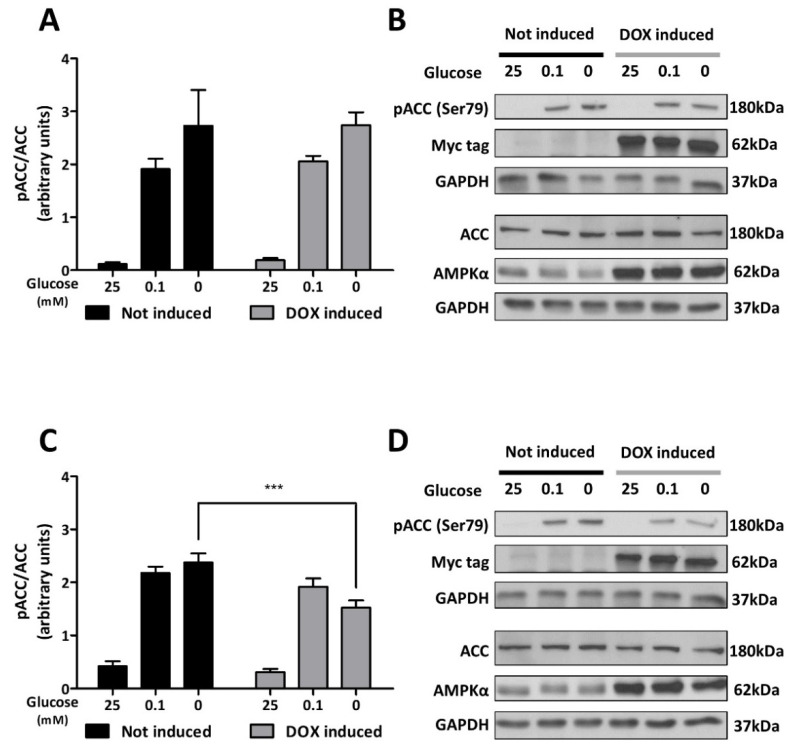
AMPK activity upon glucose deprivation in C6 cells. C6-rtTA-AMPK-DN cells induced or not with DOX (2 µg/mL, 24 h) were submitted to severe (0.1 mM) or total (0 mM) glucose deprivation for 3 (**A**,**B**) and 6 h (**C**,**D**). Phosphorylated ACC and total ACC were immunoblotted in separate gels and their expression level was normalized to GAPDH. (**A**–**C**) Histograms show means ± SEM. (**B**–**D**) Blots shown are representative of seven independent experiments. Statistical analyses were performed by two-way ANOVA followed by Bonferroni’s multiple comparison test (*** *p* < 0.001).

To characterize the impact of the partial silencing of AMPK on the metabolic plasticity of C6 cells under glucose deprivation, C6-rtTA-AMPK-DN cells were exposed to low-glucose (0.1 mM) or glucose-free media for 3 or 6 h. Immunoblots in Figure 4A show that severe or total glucose deprivation increased at least by five-fold the activity of AMPK in C6-rtTA-AMPK-DN cells not exposed to DOX. After a 6 h incubation period in the absence of glucose, cells expressing the α_1_DN showed reduced AMPK activity (37% reduction in the pACC/ACC ratio as compared to non-induced cells) (Figure 4). It is noteworthy that such reduction was not detected after only 3 h of glucose deprivation where a strong AMPK activation was observed. Studies have shown that the activation of AMPK in diverse models upon glucose deprivation is maximal after 2–3 h, before a progressive decline [43,44]. Quantification of the pACC/ACC ratio constitutes a highly sensitive readout of AMPK function. This might limit its use to estimating changes in the activity of AMPK by the DN, once triggered by a robust stress. Hence, a more efficient silencing of AMPK could be required to detect an influence on the pACC/ACC ratio after 3 h of glucose deprivation.

**Figure 5:**
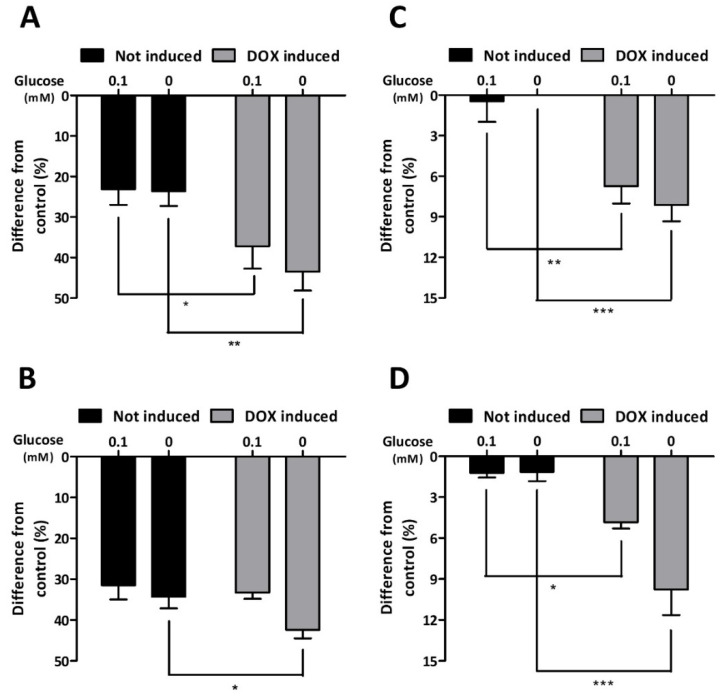
Influence of α_1_DN expression on the metabolic activity of C6 cells. C6-rtTA-AMPK-DN cells induced or not with DOX (2 µg/mL, 24 h) were submitted to severe (0.1 mM) or total (0 mM) glucose deprivation for 3 (**A**–**C**) and 6 h (**B**–**D**). Their metabolic activity and their intracellular ATP content were assessed by MTT colorimetric assay (**A**,**B**) or by a luciferase-based assay (**C**,**D**), respectively. Histograms represent the difference from the control condition (25 mM of glucose) and show means ± SEM from four (**C**,**D**) or five (**A**,**B**) independent experiments, in which every experimental condition was tested in octuplicate. Statistical analyses were performed by two-way ANOVA followed by Bonferroni’s multiple comparison test (* *p* < 0.05, ** *p* < 0.01, *** *p* < 0.001).

To further evaluate the effect of partial AMPK silencing on the capacity of C6 cells to adapt to glucose deprivation, their metabolic activity was assessed by MTT assay (Figure 5A,B), which measures the reducing capacity of viable cells [45]. Decreases in metabolic activity of 30 and 33% were measured in non-induced cells after a 6 h incubation period in low-glucose and glucose-free media, respectively. In DOX-treated cells, total glucose deprivation elicited a more pronounced decrease in metabolic activity when compared to control cells (Figure 5B). We further investigated the impact of partial AMPK silencing by measuring the intracellular content of ATP after glucose deprivation (Figure 5C,D). While non-induced cells maintained rather stable ATP content under these conditions, cells expressing the α_1_DN mutant of AMPK showed a statistically significant decrease in ATP content when exposed to severe (0.1 mM) or total (0 mM) glucose deprivation. 

**Figure 6:**
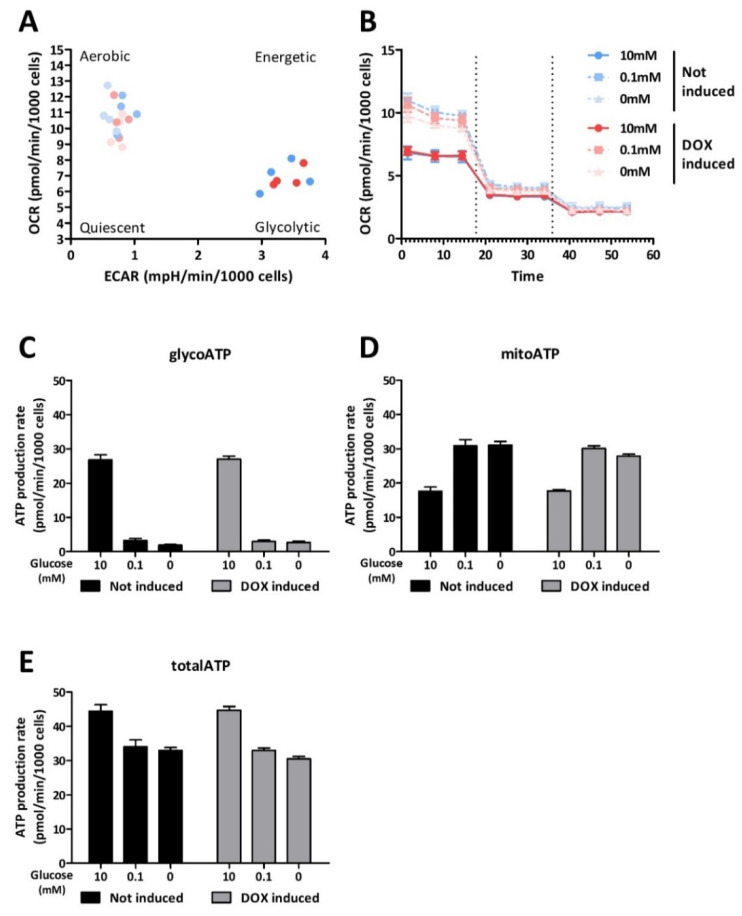
Metabolic profiling and ATP production rate of C6-rtTA-AMPK-DN cells. The oxygen consumption rate (OCR) and the extracellular acidification rate (ECAR) were measured using the Seahorse XFe96 flux analyzer. The rate of ATP production in C6-rtTA-AMPK-DN cells was quantified by real-time ATP rate assay following 6 h of severe (0.1 mM) or total (0 mM) glucose deprivation. Data from four biological replicates are presented as means ± SEM. (**A**) Energetic map of not induced (blue) and DOX-induced (red) cells in all conditions tested. All replicates are represented on the dotted plot. (**B**) Kinetic profile of the OCR measurements. (**C**,**D**,**E**) Histograms show glycolytic, mitochondrial, and total ATP production rates, respectively. Statistical analyses were performed by two-way ANOVA followed by Bonferroni’s multiple comparison test.

Further metabolic phenotyping of C6-rtTA-AMPK-DN cells undergoing glucose deprivation was obtained through the Real-Time ATP Rate Assay performed using the Seahorse metabolic flux analyzer. The Seahorse XF analyzer allows one to quantitatively assess the mitochondrial and glycolytic ATP production rates through the measures of changes in oxygen consumption and extracellular acidification, after sequential addition of the metabolic modulators oligomycin (inhibitor of complex V of the electron transport chain) and rotenone/antimycin A (inhibitors of complexes I and III of the electron transport chain, respectively). Under standard culture conditions, C6-rtTA-AMPK-DN cells mainly generated ATP through a glycolytic metabolism, and this was not significantly modified after partial silencing of the AMPK activity (Figure 6A,C). Upon complete glucose deprivation, the oxygen consumption rate increased in C6-rtTA-AMPK-DN cells, reflecting an increase in the mitochondrial ATP production rate (Figure 6B,D). The same observation was obtained 6 h after adding DOX, indicating that cells can switch to mitochondrial ATP production when glycolysis is reduced, independently of the AMPK activity (Figure 6C,D). This metabolic shift, however, cannot entirely compensate for the lack of glucose, as total ATP production rates are lower in both cell lines (Figure 6D). It is noteworthy that when treating cells with DOX, the switch to mitochondrial ATP production tends to be less efficient, and the mitochondrial ATP production rate seems slightly lower (*p* = 0.08) (Figure 6D). This suggests that the AMPK activity might contribute to the activation of mitochondria in conditions of stress.

Together, these results indicate that C6 cells adapt their metabolism to glucose deprivation. The production of ATP is reduced, but the total content of ATP is preserved, suggesting a parallel reduction in the consumption of ATP. This homeostasis is altered in cells where AMPK is partially silenced, an effect that appears not to be predominantly caused by enhanced loss of ATP production, but more likely reflects an insufficient regulation of ATP consumption.

### 3.4. AMPK Silencing Impairs Cell Migration

**Figure 7:**
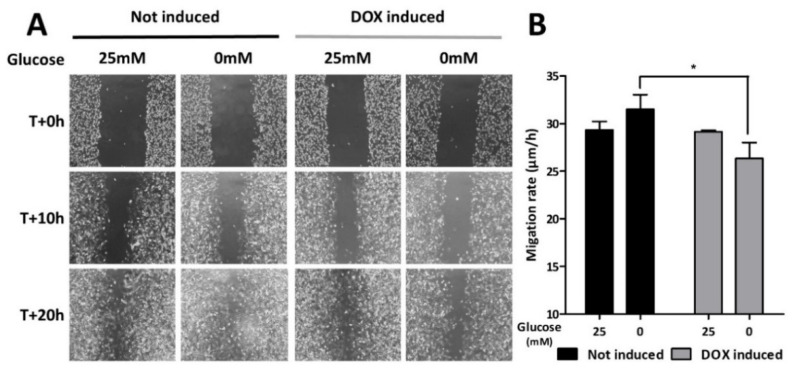
Effect of α_1_DN expression on C6 cells migration.C6-rtTA-AMPK-DN cells treated or not with DOX (2 µg/mL; 24 h) were submitted to glucose deprivation, and cell migration was examined by measuring the kinetics of cell recolonization after mechanically scratching the cell monolayer. Pictures (10× magnification) were taken at the beginning of the experiment (0 h) and every 2 h from +8 h onward over a 12 h period. (**A**) Pictures are representative of four independent experiments, in which every experimental condition was tested in triplicate. (**B**) Histograms illustrate the cell migration kinetic and represent means ± SEM. Statistical analyses were performed by two-way ANOVA followed by Bonferroni’s multiple comparison test (* *p* < 0.05).

Even though astrocytes are mostly quiescent and non-migratory cells in physiological conditions, it has been shown that in response to diverse cellular stresses, reactive astrocytes show increased mobility. To characterize the role of AMPK in the regulation of such adaptation, a migration assay was conducted in C6-rtTA-AMPK-DN cells submitted to metabolic stress. The migration of the cells was studied in vitro by examining the cell colonization within a wound that was mechanically realized in a confluent cell monolayer. As expected, glucose deprivation elicited a modest increase in C6 motility. Induction of the α_1_DN expression with DOX did not affect cell motility in standard glucose conditions. However, under glucose deprivation, the cell colonization within the wound area was significantly reduced compared to non-induced cells (Figure 7). This data indicates that AMPK participates in the regulation of C6 cell migration in conditions of glucose deprivation, suggesting its role in the adaptation of these cells to stress.

### 3.5. AMPK Silencing Does Not Alter Glutamate Transport

**Figure 8:**
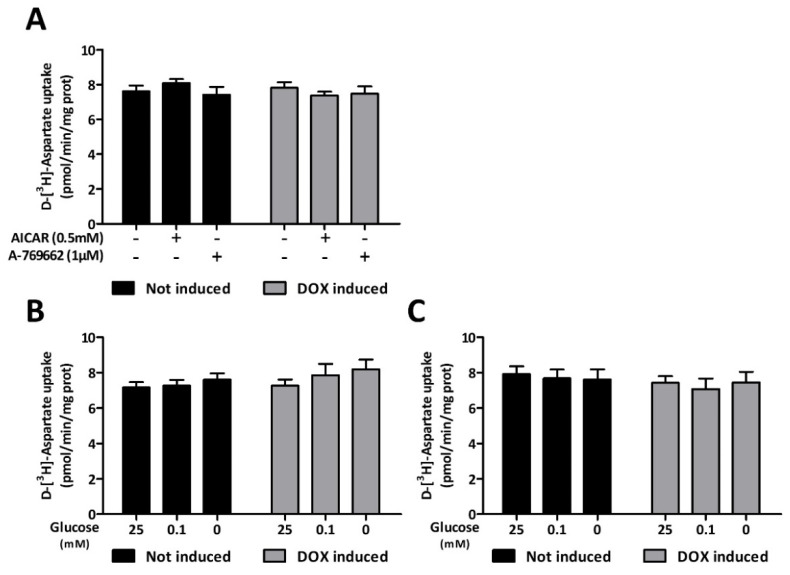
AMPK regulation of glutamate transport. The activity of glutamate transporters was evaluated by measuring the uptake of d-[^3^H]-aspartate in C6-rtTA-AMPK-DN cells exposed or not to DOX (2 µg/mL; 24 h). (**A**) C6-rtTA-AMPK-DN cells were either treated with the pharmacological AMPK activators AICAR (0.5 mM) and A-769662 (1 µM) for 3 h, or (**B**,**C**) exposed to glucose deprivation conditions for 3 and 6 h. Histograms show means ± SEM of data obtained from three independent experiments performed in quadruplicate. Statistical analyses were performed by two-way ANOVA followed by Bonferroni’s multiple comparison test.

The uptake of glutamate by astrocytes indirectly consumes ATP. In addition, during periods of intense synaptic activity, this uptake and the associated Na^+^ flux serves as a biochemical signal that triggers the metabolic adaptations of these glial cells [9,14,46]. To investigate the influence of AMPK on the regulation of the activity of glutamate transporters, C6-rtTA-AMPK-DN cells were treated with AMPK activators or exposed to glucose deprivation before monitoring the uptake of radiolabeled aspartate. As shown in Figure 8A, activation of AMPK with either AICAR (0.5 mM) or A-769662 (1 µM) for 3 h was without influence on the aspartate uptake velocity. This suggests the absence of direct modulation of glutamate transporters by this kinase. Similarly, submitting the cells to severe (0.1 mM) or total glucose deprivation for up to 6 h did not influence the substrate uptake. Such resilience was not supported by the activity of AMPK, as its partial silencing upon DOX treatment did not cause any significant loss of aspartate uptake in all tested conditions of glucose deprivation (Figure 8B,C).

## 4. Discussion

The objective of the present study was to examine the implication of AMPK in the adaptation of astrocyte functions in response to a metabolic stress. This was specifically examined by manipulating AMPK activity in a model of astrocytoma cells maintained in glucose containing medium or in conditions of glucose deprivation. Considering the difficulty in achieving efficient and specific pharmacological inhibition of AMPK [47], a genetic approach was used to silence AMPK. A dominant negative mutant of the α_1_ subunit of AMPK was obtained by introducing a mutation at position 157 to generate an inactive α subunit that is thought to compete with both endogenous α isoforms for β and γ, causing a silencing of both α_1_- and α_2_-containing complexes [48]. Avoiding any bias related to the comparison of different transfected cell populations, a single cell clone was used, which carries a DOX-inducible system allowing one to manipulate the expression level of the α_1_DN. The study was performed in a model of C6 rat astrocytoma cells [35] that can easily be genetically manipulated. Even though their use presents limitations because of their malignant origin, a comparative study between C6 cells and primary cultures of astrocytes has shown that these cells respond similarly to certain stimuli [49]. Moreover, accumulating evidence states that astrocyte-derived tumors actually retain or exacerbate many key features of “normal” astrocytes, notably their metabolic plasticity [50]. 

To study their adaptation to a metabolic stress, C6 cells were submitted to severe (0.1 mM) or total (0 mM) glucose deprivation for 6 h. This differs from several other studies where cells were exposed to transient deprivation of both glucose and oxygen as an in vitro model of stroke to specifically study ischemic cell death [51]. Considering the rapid reversibility of the inducible system, DOX was maintained in the culture medium at all times, even during the deprivation step. The metabolic profiling of the cells was first examined using the Seahorse XF analyzer, which allows one to simultaneously measure the glycolytic and mitochondrial ATP production in living cells in order to appreciate the contributions of both catabolic pathways. In standard culture conditions, C6 cells showed a predominant glycolytic phenotype, which is consistent with their astrocytic nature [14,52,53]. When deprived of glucose, the rate of ATP production was reduced, which was clearly assigned to a considerable reduction in glycolysis. Even though this was accompanied by a robust increase in mitochondrial ATP production, this metabolic switch could not fully compensate the loss in glycolytic ATP. The altered metabolic profile of C6 cells maintained in the absence of glucose was also evident in the MTT assay, revealing the vulnerability of the cells in these conditions. Strikingly, however, the total ATP content in C6 cells was found to remain stable under stress conditions, suggesting that a decrease in energy consumption paralleled the decrease in energy production to maintain homeostasis. Remarkably, glucose deprivation was found to trigger the activation of AMPK in C6 cells, as evidenced by the increase in the phosphorylation of its specific endogenous substrate ACC, thus confirming its role as a metabolic sensor in these cells. Together, these results highlight the resilience of these astrocyte-like cells towards metabolic stress and suggest the implication of AMPK in their metabolic adaptation.

In astrocytes, the detection of both α isoforms of AMPK has been reported [27,28]. However, no other study has specifically detailed the relative quantification of these AMPK isoforms in glial cells. The expression profile of endogenous AMPKα mRNAs was herein evaluated in a model of C6-rtTA cells, in which we found a predominant expression of the α_1_ isoform. These observations are consistent with studies focusing on cancer cells in which the *Prkaa1* mRNA is commonly detected [25,47], notably in high-grade astrocytoma [54]. Moreover, high-metabolic-demand tissues, such as the liver or the kidneys, are also characterized by an abundant expression of the α_1_ isoform [21]. In the brain, Meares et al. have reported on the immunodetection of both α isoforms, with a predominant expression of the α_1_ isoform in astrocytes [55]. These observations are in accordance with our recent findings on primary cultures of astrocytes when comparing AMPKα isoform expression by qPCR (Belo do Nascimento et al., in preparation).

Using the inducible system, we were able to manipulate the expression of the α_1_DN as evidenced by a robust increase in the *Prkaa1* mRNA expression after exposure to DOX. The induction was also validated by immunoblotting, further confirming that the expression of the recombinant protein was DOX concentration dependent. It has been suggested that overexpression of α_1_DN destabilizes the endogenous AMPK complexes and promotes the degradation of both endogenous AMPKα_1_ and AMPKα_2_ [48]. Even though this possibility was not formally addressed, since both the endogenous α_1_ and α_1_DN isoforms are recognized by the same antibodies, we found that the endogenous *Prkaa1* mRNA expression was not altered by overexpression of the α_1_DN. This suggests that DOX treatment has no effect on the endogenous expression of AMPK. Besides validating the inducible expression of the DN, the efficacy of the functional silencing of AMPK was assessed by measuring its kinase activity in response to a stimulus. Overexpression of the α_1_DN partially reduced the AMPK activity triggered by two biochemical AMPK activators, as illustrated by a lower pACC/ACC ratio when compared to control cells.

A major observation regarding the impact of silencing AMPK on the metabolic adaptation of C6 cells is the inability of α_1_DN-expressing cells to maintain stable ATP levels under glucose deprivation. This was observed at both time points tested, after severe or total glucose deprivation. Similarly, these cells showed an increased vulnerability to metabolic stress, as indicated by their reduced activity measured in the MTT assay. Data from both ATP and MTT assays could reflect a reduction in cell number due to reduced viability after exposure to stress conditions. Nevertheless, it is noteworthy that silencing AMPK activity was shown to exacerbate these outcomes, confirming their vulnerability. The compromised metabolic adaptation of DOX-induced C6 cells was also noticeable in a cell migration assay. Several studies have indeed suggested that enhanced astrocyte mobility represents an adaptive mechanism in response to energetic stress [56,57]. In the present study, even if silencing of AMPK had no effect on cell migration in standard glucose conditions, a decreased cell motility in response to glucose deprivation was observed in α_1_DN-expressing cells.

The compromised adaptive properties observed in α_1_DN-expressing cells are consistent with their limited capacity to activate AMPK-dependent signaling pathways, as also revealed by a reduced phosphorylation of the ACC, here used as an indicator of the kinase activity. Preservation of the energetic homeostasis constitutes the major role of AMPK in conditions of stress. The observation that α_1_DN-expressing cells are less capable of adapting to glucose stress conditions therefore highlights the importance of AMPK in C6 cells’ metabolism. The decrease in total ATP levels herein observed when combining glucose deprivation and AMPK silencing is indicative of an altered balance between ATP-consuming and -generating processes. As observed in non-induced cells, metabolic profiling of the DOX-induced C6 cells submitted to glucose deprivation actually revealed a similar switch towards mitochondrial activity, despite the partial silencing of AMPK. This suggests that the loss of ATP homeostasis does not result from a decreased production of ATP, but rather from a failure of the cell to repress ATP-consuming processes to save energy.

AMPK activation has been suggested to regulate several ion channels and transporters [58], but there is only limited evidence for a regulation of monoamine or amino acid transporters by this enzyme. It is, however, noteworthy that the co-transport of Na^+^ constitutes the driving force supporting the uptake of specific substrates against their concentration gradient, and that the extrusion of Na^+^ consumes substantial amount of ATP. In a model of Xenopus oocytes expressing the glutamate transporters EAAT3 or EAAT4, co-expression of constitutively active mutants of AMPK was shown to downregulate Na^+^-coupled glutamate transport [59]. The molecular mechanism has not been elucidated in this study, but the implication of the ubiquitin ligase Nedd4-2 was evidenced, and the direct phosphorylation of the EAAT transporter by AMPK was proposed. Even though EAAT3 is expressed in C6 cells [60], our present work failed to provide evidence of an alteration in the glutamate uptake after pharmacological activation of AMPK. These observations suggest that the transport of glutamate is not directly regulated by AMPK. Similar observations were reported by Voss et al. in cultured astrocytes, showing that the pharmacological activation of AMPK was without influence on glutamate uptake or its conversion to glutamine [61]. Furthermore, in their follow-up study in 2020, the authors found that in hippocampal slices, AMPK activation did not alter glutamate uptake nor its entry in the TCA cycle [29].

Considering the herein demonstrated role of AMPK in preserving ATP homeostasis in C6 cells in conditions of metabolic stress, the silencing of AMPK could indirectly influence the glutamate uptake capacity. Indeed, the loss of ATP could result in local alterations in the Na^+^-gradient that support efficient uptake. Our data show that even in condition of glucose deprivation, the uptake of glutamate was not altered in DN-expressing C6 cells. This indicates either that the uptake of glutamate is highly resistant to metabolic stress in C6 cells or that the inhibition of AMPK obtained with the dominant negative approach is insufficient to affect this essential astrocytic function.

## 5. Conclusions

The present study provides evidence for the functional resistance of C6 cells when facing a metabolic stress, herein modeled by glucose deprivation. Such resilience is supported by AMPK likely regulating the balance between the energy-producing and -consuming processes. Indeed, in tissues showing a high metabolic rate, such as muscle or liver, metabolic stresses promote the activity of AMPKs that coordinate a program of energy preservation. This includes the enhancement of ATP-generating processes and the inhibition of energy-consuming or -storing pathways, such as lipid, glycogen, and protein synthesis. Glutamate uptake, which indirectly consumes ATP, was found to be unaltered in conditions of metabolic stress, even when AMPK was partially silenced. As an essential astrocytic function in the support of neuronal activities, one may hypothesize that the glutamate uptake is not affected by adaptive processes operating in astrocytes exposed to stress. Hence, some recent studies have shown that glutamate, which may serve as an alternative energetic substrate for glial cells, is sufficient to pay the costs of glutamate uptake [13].

Besides oxidative stress and neuroinflammation, glutamate excitotoxicity and dysregulation of energetic homeostasis are commonly implicated in the development or progression of several neurological diseases such as Alzheimer’s disease, amyotrophic lateral sclerosis, and chronic/neuropathic pain. Largely contributing to the protection of neurons against these cellular insults, astrocytes are commonly pointed out as key actors in these diseases. Recent studies have proposed AMPK as a promising therapeutic target for neurodegenerative diseases [31]. The need to inhibit or reinforce AMPK activity remains a question of debate, and in this context, the absence of influence of AMPK on the activity of glutamate transporters could be considered as an advantage.

## Figures and Tables

**Table 1:** RT-qPCR primer sequences.

Gene	Forward Sequence (5′–3′)	Reverse Sequence (5′–3′)
*Prkaa1*	TTAAACCCACAGAAATCCAAACAC	CTTCGCACACGCAAATAATAGG
Endogenous *Prkaa1*	ATGCGCAGACTCAGTTCCTG	GTCCAGTCAACTCGTGCTTG
*Prkaa2*	GTGGTGTTATCCTGTATGCCCTTCT	CTGTTTAAACCATTCATGCTCTCGT
*Gapdh*	GTCTCCTGTGACTTCAACAG	AGTTGTCATTGAGAGCAATGC

## Data Availability

The data presented in this study are available from the corresponding author on request.
